# Supplementary material for: The activity, divergence, and evolutionary degradation of modern-day homing endonucleases and their reconstructed ancestors
Source: bioRxiv. 2026 Jul 14:2026.07.13.738334. Preprint. [Version 1] doi: 10.64898/2026.07.13.738334 (PMC13405050; doi:10.64898/2026.07.13.738334)
Supplement: 1 [file NIHPP2026.07.13.738334v1-supplement-1.pdf]

bioRxiv preprint doi: https://doi.org/10.64898/2026.07.13.738334; this version posted July 14, 2026. The copyright holder for this preprint (which was not certified by peer review) is the author/funder, who has granted bioRxiv a license to display the preprint in perpetuity. It is made available under a CC-BY 4.0 International license.

Supplementary Material

The activity, divergence, and evolutionary degradation of modern-day homing endonucleases and their reconstructed ancestors

Juliana C. Young[1,#], Abigail R. Lambert[1,#], Janet M. Young[1], Lindsey A. Doyle, Miriam Silverstein[1], David R. Edgell[2,*] and Barry L. Stoddard[1,*]

[1] Division of Basic Sciences

Fred Hutchinson Cancer Research Center

1100 Fairview Ave. North, Seattle WA 98109 USA

[2] Department of Biochemistry

Schulich School of Medicine and Dentistry

University of Western Ontario

London, ON N6A 5C1, Canada

[#] Co-first authors

[*] Corresponding author: bstoddar@fredhutch.org

bioRxiv preprint doi: https://doi.org/10.64898/2026.07.13.738334; this version posted July 14, 2026. The copyright holder for this preprint (which was not certified by peer review) is the author/funder, who has granted bioRxiv a license to display the preprint in perpetuity. It is made available under a CC-BY 4.0 International license.

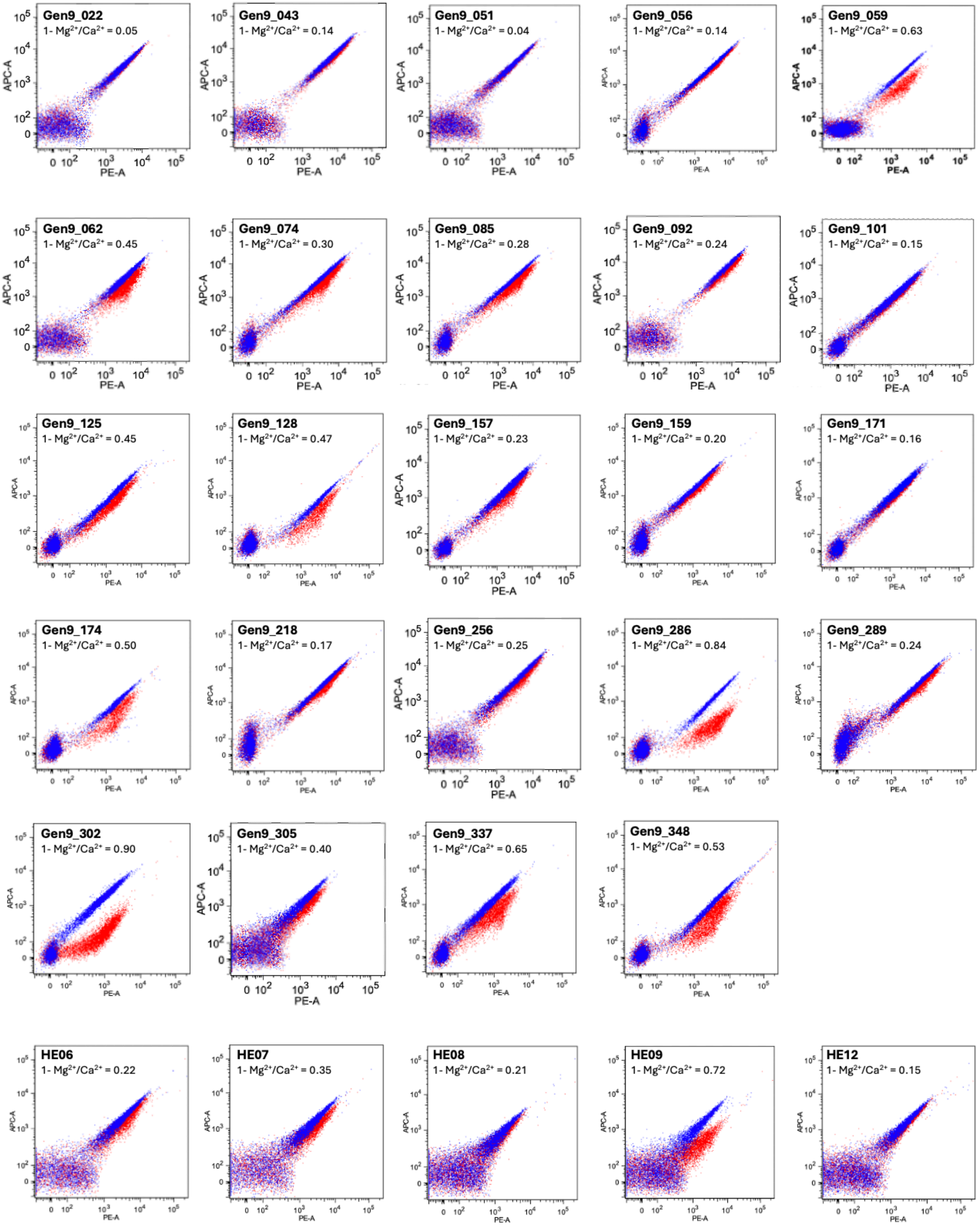

bioRxiv preprint doi: https://doi.org/10.64898/2026.07.13.738334; this version posted July 14, 2026. The copyright holder for this preprint (which was not certified by peer review) is the author/funder, who has granted bioRxiv a license to display the preprint in perpetuity. It is made available under a CC-BY 4.0 International license.

Supplementary Figure S1. Tethered Flow Cytometric Cleavage Assay Data for 29 Active LHEs. Flow cytometric plots are shown for the 29 LHEs determined to be active against their predicted DNA target sites. Each plot is a superposition of yeast with tethered DNA target substrate in the presence of buffer with Ca^{2+} (blue, no cleavage of the tethered DNA) or in the presence of buffer with Mg^{2+} (red, cleavage of the tethered DNA). When target cleavage occurs, the cut portion of the substrate containing the A647 tag is washed away, resulting in a drop in A647 signal. If the LHE fails to release the DNA after cleavage has occurred (referred to as “end-holding”), there will be no drop in A647 signal. The quantified cleavage values (1 – Mg^{2+}/Ca^{2+} Ratio) are listed for each enzyme, with a threshold activity value of greater than 0.1 used to designate active enzymes. Two of the 29 active LHEs (Gen9-022 and Gen9-051) had quantified activity values less than the 0.1 cutoff, but showed activity in the complementary non-tethered in vitro cleavage assay.

bioRxiv preprint doi: https://doi.org/10.64898/2026.07.13.738334; this version posted July 14, 2026. The copyright holder for this preprint (which was not certified by peer review) is the author/funder, who has granted bioRxiv a license to display the preprint in perpetuity. It is made available under a CC-BY 4.0 International license.

a

Non-Tethered DNA Cleavage Activity Assay

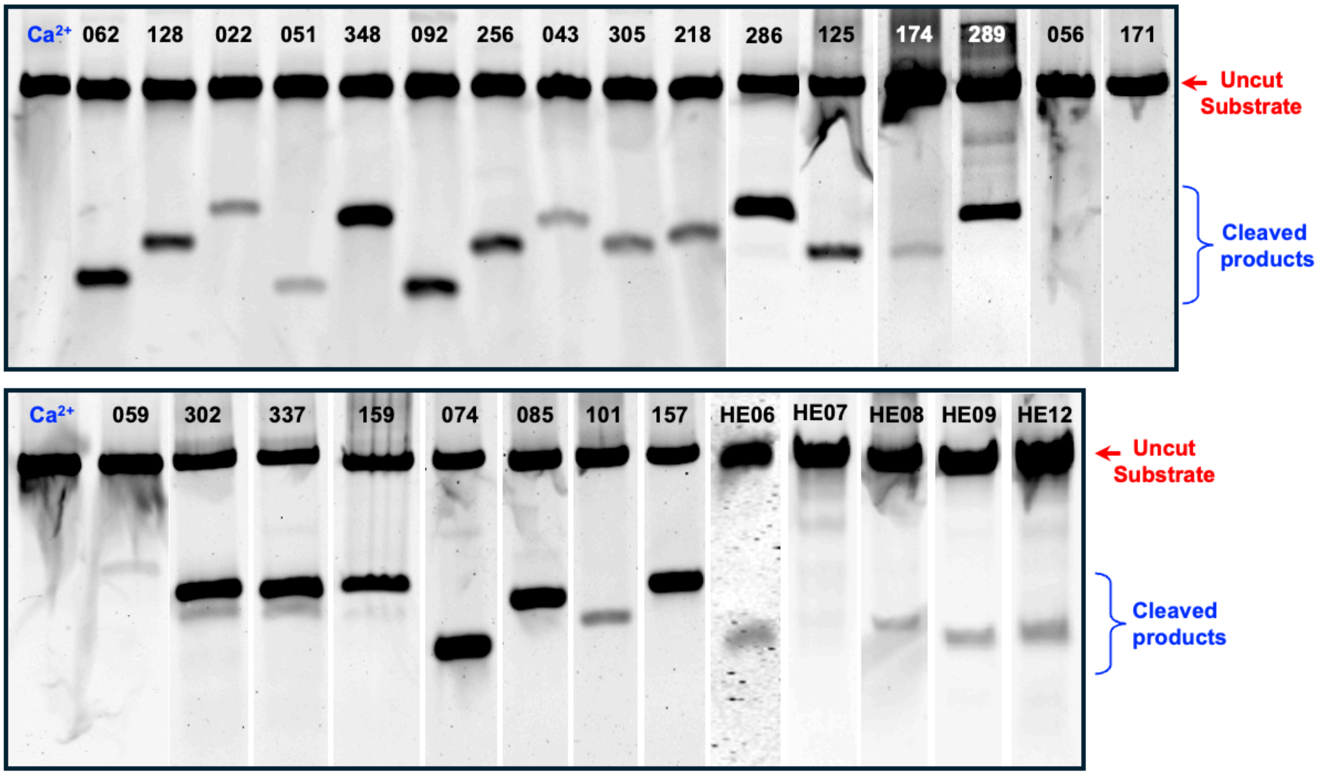

b

Target sites with central four basepairs verified (shown as 22bp target sites)

```
022   ACACGATACTTACTATGTAGTT      286   GCTGCTTCTAAGCTAACCTCCT
043   TCTTCGGTCACCCTGAAGTTTA      305   GGTTGGACGGTATATCCACCAC
059   TCTTCGGGCATCCCGAGGTAAA      337   GGATGGACAGTATATCCTCCTT
062   AACCTCCCCAGATGAATTCTAC      348   GTGATCCTCATAATCAAAGGTT
085   GTTGAGTTCATTTGAGGAGGTT      HE06  GGTTGAACTCTTTATCCTCCTT
092   GAGGTCCTGATATGGCATTCCC      HE07  ATATACATTATGAGGTTCTTTA
128   GTGGAGTAGGTACAGGATGGAC      HE09  TTGATTCATATATGTTGCCTGA
157   TTACCAAAAATATCTGAGCATG      HE12  TTCCATAGTGCATTAACACCTA
174   GGGTACAGGGTTCCATGGAATT
218   TGGATTTGGTAACTATATTCTA
256   GGTTGAACTCTTTACCCACCAT
```

c

Target regions with central four basepairs not verified (shown as 58bp target regions)

```
HE08  ACAGTATTCAATTGGATATATGAGCCATGATCCTCACAATCAAAGATTCTTTAGTTAT
 051  TGATCCAATCTTATATCAACACTTATTCTGGTTCTTTGGACACCCAGAAGTTAAAAAT
 056  AGGAACGAAGGCATTGCGTCACGAAAAGGACTAGGGACCCTAGTAGTCTTTGCAGAAA
 074  TGATCCATTATTATACCAACATTTATTCTGGTTCTTTGGACATCCAGAAGTTTATATT
 101  GACTGGTTATACCACTTAAGGGTGGATAGACCGTCCACCCTGAACCGCACATGGAAGC
 125  TTATTCTGATTCTTTGGACATCCAGAGGTTTATATTCTAATAGTACCAGGATTTGGTA
 159  TATTCCAACACCTCTTCTGGTTCTTCGGTCACCCAGAAGTCTACATTATGATTTTACC
 171  AAGAAAAGGAGATAGAAGCACCCAGAAGTTTATATCTTAATTATCCCTGGATTTGGTA
 289  GAAGAAGGACCAGGAACAGGTTGAACAGTTTATCCACCACTATCAGGAATACAATCAC
 302  GAGACAGGACCAGGGACAGGGTGAACTGTATACCCACCTCTTTCAAGTATCCAATCAC
```

bioRxiv preprint doi: https://doi.org/10.64898/2026.07.13.738334; this version posted July 14, 2026. The copyright holder for this preprint (which was not certified by peer review) is the author/funder, who has granted bioRxiv a license to display the preprint in perpetuity. It is made available under a CC-BY 4.0 International license.

Supplementary Figure S2. Cleavage activity in the non-tethered in vitro DNA cleavage assay for 29 active LHEs. (A) Gel electrophoresis of cleavage products from the non-tethered in vitro cleavage assay, imaged on a Typhoon fluorescent imager. DNA bands are visible due to the presence of the fluorescent A647 tag. Images of gels from separate experiments on multiple days are combined into one image. The red arrow indicates the location of the uncut DNA target substrates, which aligns with the lane containing the Ca^{2+} uncut control. Blue brackets indicate the location of successfully cleaved DNA fragments. The position of the cut within each 58bp target site substrate was not necessarily in the center of the DNA substrate, so the cleaved products are not all the same size. Three of the active enzymes were presumed to be weak binders (Gen9-056, Gen9-171, HE07), as they did not produce cleavage products in this assay even though they showed cleavage in the tethered flow cytometric cleavage assay. **(B)** Centered 22 base pair target sites of the active enzymes for which the precise location of cleavage on each DNA strand could be determined using run-off sequencing. The central four basepairs (which are flanked by the sites of phosphoryl hydrolysis on each individual DNA strand, producing corresponding 4-base, 3’ overhangs from those basepairs) are indicated in blue. **(C)** For those LHEs where the precise center of the DNA target sequences could not be determined (inability to isolate sufficient amounts of gel-purified products or failure to obtain clean run-off sequencing data), the sequence of the full 58 bp DNA target substrate is shown. The purple and red bases correspond to the flanking 5’ and 3’ exon sequences that were joined together to produce the predicted target site region for each candidate LHE. The site of cleavage occurs somewhere within these 58bp DNA sequences.

bioRxiv preprint doi: https://doi.org/10.64898/2026.07.13.738334; this version posted July 14, 2026. The copyright holder for this preprint (which was not certified by peer review) is the author/funder, who has granted bioRxiv a license to display the preprint in perpetuity. It is made available under a CC-BY 4.0 International license.

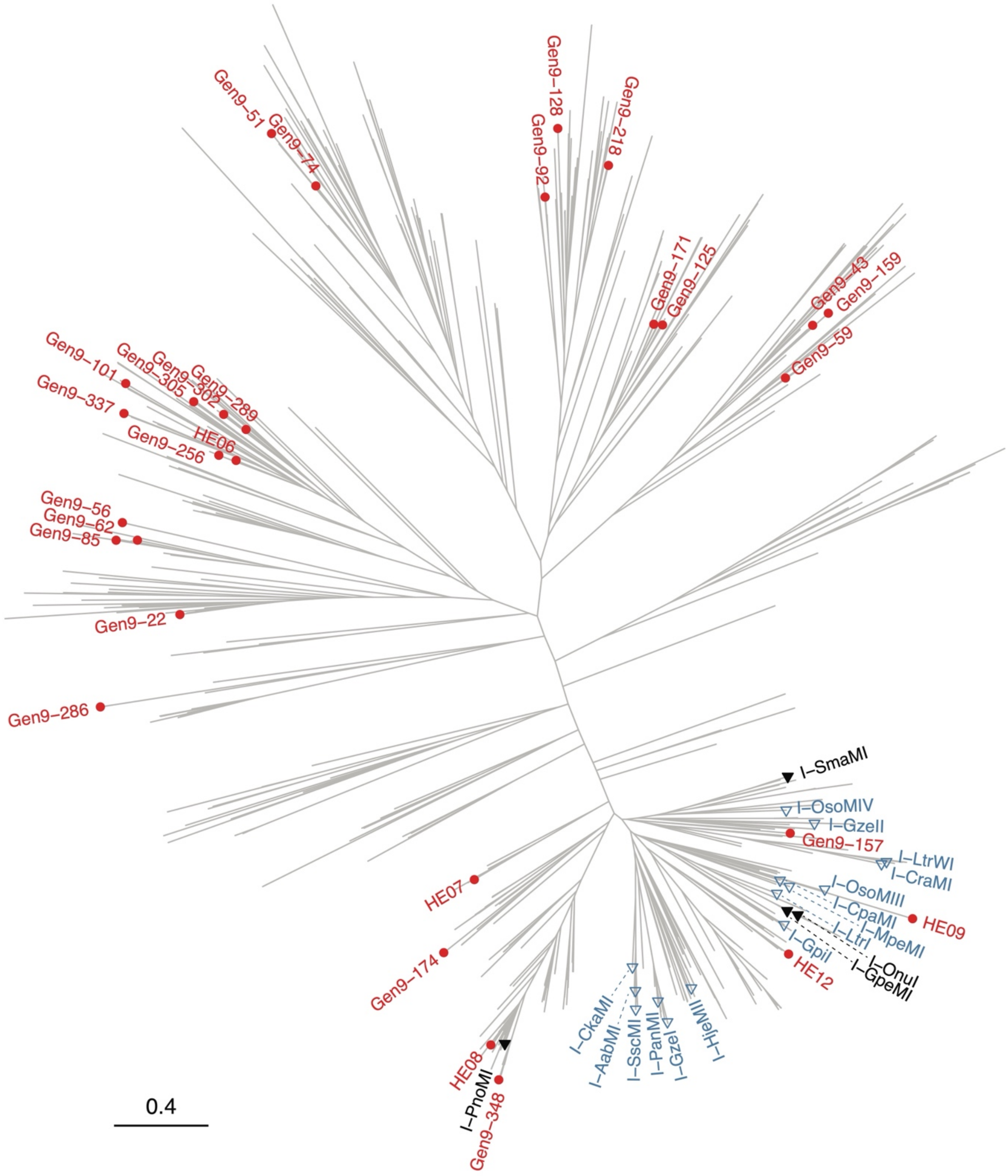

Supplementary Figure S3. Phylogenetic tree of expanded set of LHEs. We aligned our larger 2023 dataset of 622 LHE sequences together with 11 sequences from the original experimentally tested dataset that were inadvertently missing from the 2023 set, making a total of 633 sequences. We aligned all 633 sequences using MAFFT, and removed alignment positions where >50% of sequences contained a gap. In the resulting alignment, two sequences were quite truncated and were removed before we estimated a phylogeny using PHYML, displayed using R and the ggtree package. See Methods for full details. Red labeled dots indicate the 29 newly identified active LHEs, and blue/black labeled triangles indicate 19 previously characterized LHEs, with selected sequences used in ancestral reconstruction highlighted in black.

bioRxiv preprint doi: https://doi.org/10.64898/2026.07.13.738334; this version posted July 14, 2026. The copyright holder for this preprint (which was not certified by peer review) is the author/funder, who has granted bioRxiv a license to display the preprint in perpetuity. It is made available under a CC-BY 4.0 International license.

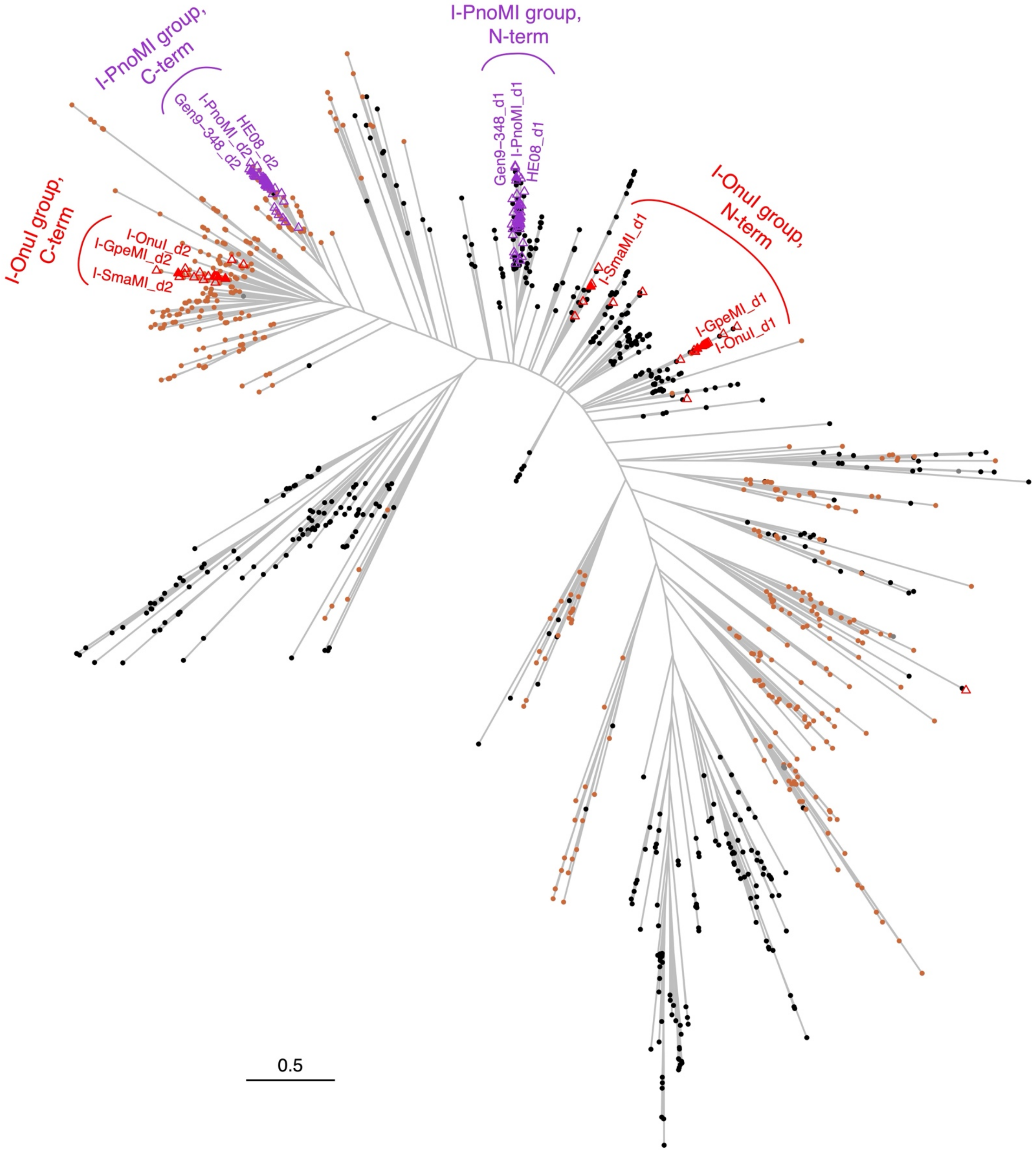

bioRxiv preprint doi: https://doi.org/10.64898/2026.07.13.738334; this version posted July 14, 2026. The copyright holder for this preprint (which was not certified by peer review) is the author/funder, who has granted bioRxiv a license to display the preprint in perpetuity. It is made available under a CC-BY 4.0 International license.

Supplementary Figure S4. Phylogenetic tree of all individual LADLIDADG domains extracted from the dataset of 622 candidate LHEs. We used the hmmsearch tool to identify and align all matches to PFAM's for the "LAGLIDADG_1" domain Hidden Markov Model (PF00961.22, 102aa long). Filtering to remove apparently truncated domain sequences (matching <75 aa of the domain HMM) resulted in an alignment of 1140 domains from 610 candidate LHEs. We estimated a maximum likelihood tree using PHYML and used the ggtree R package to display the tree with annotations. Almost all sequences contained two LAGLIDADG domain matches: black dots at tips of the tree are N-terminal domains, and brown dots are C-terminal domains. The scale bar indicates number of amino acid substitutions per site. We used this tree to select candidate LHEs for ancestral reconstructions for each of two sequence groups of interest: (a) I-PnoMI, Gen9-348 and HE08 (purple) and (b) I-OnuI and I-GpeMI (red). For each of the two sequence domains in each of the two groups, we located the focal members (purple/red filled triangles and text labels, with "_d1" and "_d2" indicating the N- and C-terminal domains, respectively) and identified their most recent common ancestral node. We stepped back three nodes deeper in the tree to recruit some outgroup sequences, and extracted a list of all descendants of that deeper node. We then merged the lists from the two domains in each group, and used the resulting merged sequence lists (purple/red triangles) as the bases for ancestral reconstructions. Complete details are provided in the Methods section. The selected group members for the I-PnoMI group (purple) are found very near one another on the tree for both the N- and C-terminal domains, as expected. However, for the I-OnuI group, only the C-terminal domains group closely, whereas the N-terminal domains are more scattered in the tree. This pattern is likely due to incongruent evolutionary histories between the two domains, perhaps due to recombination/gene conversion: exploring that question further is beyond the scope of this study.

bioRxiv preprint doi: https://doi.org/10.64898/2026.07.13.738334; this version posted July 14, 2026. The copyright holder for this preprint (which was not certified by peer review) is the author/funder, who has granted bioRxiv a license to display the preprint in perpetuity. It is made available under a CC-BY 4.0 International license.

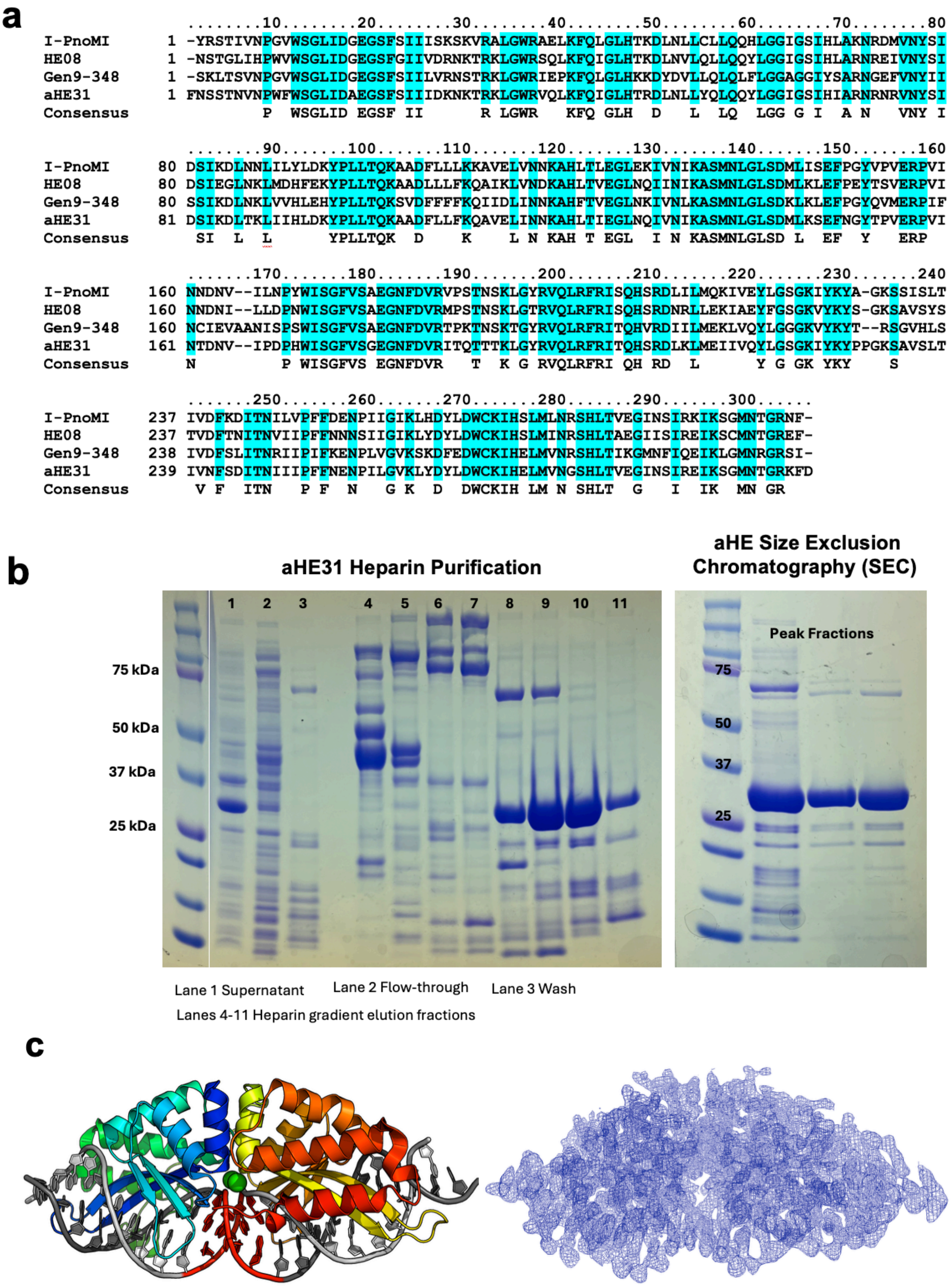

bioRxiv preprint doi: https://doi.org/10.64898/2026.07.13.738334; this version posted July 14, 2026. The copyright holder for this preprint (which was not certified by peer review) is the author/funder, who has granted bioRxiv a license to display the preprint in perpetuity. It is made available under a CC-BY 4.0 International license.

Supplementary Figure S5. Amino acid sequence alignment of the characterized I-PnoMI clade enzymes with reconstructed ancestor aHE31 and purification of recombinant aHE31 protein. **(A)** Fully conserved positions are highlighted in cyan, with the conserved sidechains listed below the alignment. **(B)** Recombinant aHE31 protein was purified using a two-step protocol, including heparin purification followed by size exclusion chromatography (SEC). The contents of lanes on the gel from the heparin purification are listed below the gel, and the aHE31 protein is expected at a molecular weight of 34.7 kDa. **(C)** The crystal structure of the aHE31 ancestral reconstruction, bound to the I-PnoMI DNA target site, was solved to 2.17 Å resolution. See **Table 1** for data and refinement statistics.

bioRxiv preprint doi: https://doi.org/10.64898/2026.07.13.738334; this version posted July 14, 2026. The copyright holder for this preprint (which was not certified by peer review) is the author/funder, who has granted bioRxiv a license to display the preprint in perpetuity. It is made available under a CC-BY 4.0 International license.

a

```
              ....:...10....:...20....:...30....:...40....:...50....:...60....:...70....:...80
I-OnuI      1 --SRRESINPWILTGFADAEGSFLLRIRNNNKSSVGYSTELGFQITLHNKDKSILENIQSTWK-VGVIANSGDNAVSLKV
I-GpeMI     1 -PTRNESINPWVLTGFADAEGSFILRIRNNNKSSAGYSTELGFQITLHKKDKSILENIQSTWK-VGVIANSGDNAVSLKV
I-SmaMI     1 SKGENSKLNPWAVVGFIDAEGSFMVRVRKNSKYKTGWLVVAIFSVTVDKKDLFLLESLKTFFGGLGSIKKSGNSTFSYRI
aHE15       1 NSSRNSSLNPWVVTGFVDAEGSFMISVRKNNKSSTGWSTQLRFQISLHKKDRSLLEQIQSYFG-VGSIRKSGDNSVSFRI
aHE11       1 SASRNESINPWVLTGFADAEGSFLLRIRNNNKSSVGYSTELGFQITLHKKDKSILENIQSTWK-VGVIANSGDNAVSLKV
consensus            iNPW   GF DAEGSFl   R N K    Gy     F  t   KD  iLE i s w    G I  SG    S k

              ....:...90....:..100....:..110....:..120....:..130....:..140....:..150....:..160
I-OnuI     78 TRFEDLK-VIIDHFEKYPLITQKLGDYMLFKQAFCVMENKEHLKINGIKELVRIKAKLNWGLTDELKKAFPEI-------
I-GpeMI    79 TRFEDLR-VVLNHFEKYPLITQKLGDYLLFKQAFSVMENKEHLKIEGIKRLVGIKANLNWGLTDELKEAFVASGGENIFV
I-SmaMI    81 ESSEQLTKIILPFFDKYSLITEKLGDYLLFKKVLELMGTKEHLTQRGLEKIVSLKASINKGLSEELQAAFPQCVPT----
aHE15      80 ESLEDLK-VVINHFDKYPLITQKHGDYLLFKQAFELMKNKEHLTIEGLKKIVAIKASLNKGLSDELKEAFPDIVPV----
aHE11      80 TRFEDLK-VVINHFEKYPLITQKLGDYLLFKQAFSVMENKEHLKIEGIKRLVGIKANLNWGLTDELKEAFPEI-------
consensus        E L     i   FeKY LIT K GDYlLFK      M  KEHL   Gi   lV iKA lN GLtdEL   AF

              ....:..170....:..180....:..190....:..200....:..210....:..220....:..230....:..240
I-OnuI    150 -ISKERSLINKNIPNFKWLAGFTSGEGCFFVNLIKS-KSKLGVQVQLVFSITQHIKDKNLMNSLITYLGCGYIKEKNKSE
I-GpeMI   158 ASGGERSLINKNIPNSGWLAGFTSGEGCFFVSLIKS-KSKLGVQVQLVFSITQHARDRELMDNLVTYLGCGYIKEKKKSE
I-SmaMI   157 ---PRPEINNKNIPDPFWLAGFVSGDGSFKSILKKSESIKVGFQSILVFQITQHARDVKLMESLISYLGCGFIEKDS--R
aHE15     155 ---TRPLVENKTIPDPEWLAGFTSGEGCFFITISKSPSSKLGVQVQLVFSLTQHTRDEALMNSLISYLGCGNIKIKKNSK
aHE11     152 -ISKERSLINKNIPNSKWLAGFTSGEGCFFVSLIKS-KSKLGVQVQLVFSITQHARDRALMNSLITYLGCGYIKEKKKSE
consensus             NK IP   WLAGF SGeG F   l KS   K G Q  LVF iTQH rD  LM  L tYLGCG I

              ....:..250....:..260....:..270....:..280....:..290....:..300....:..310..
I-OnuI    228 FSWLDFVVTKFSDINDKIIPVFQENTLIGVKLEDFEDWCKVAKLIEEKKHLTESGLDEIKKIKLNMNKGR--
I-GpeMI   237 FSWLEFVVTKFSDIKDKIIPVFQENNIIGVKLEDFEDWCKVAKLIEEKKHLTESGLEEIRNIKLNMNKGRVL
I-SmaMI   232 GPWLYYTVTNFSDIQGKIIPFFHQYKIIGSKYGDYQDWCKIALIMQNKNHLTPEGLNEIRALKGGMNKGRL-
aHE15     232 NSWLDFVVTKFSDINEKIIPFFNQHKILGVKSQDFEDWCKAAELIKDKKHLTPEGLDEIRKIKAGMNKGR--
aHE11     230 FSWLDFVVTKFSDINDKIIPVFQENNIIGVKLEDFEDWCKVAKLIEEKKHLTESGLDEIRKIKLNMNKGR--
consensus          WL f VT FSDI  KIIP F    iiG K  Df DWCK A li  K HLT  GL EIr iK  MNKGR
```

b

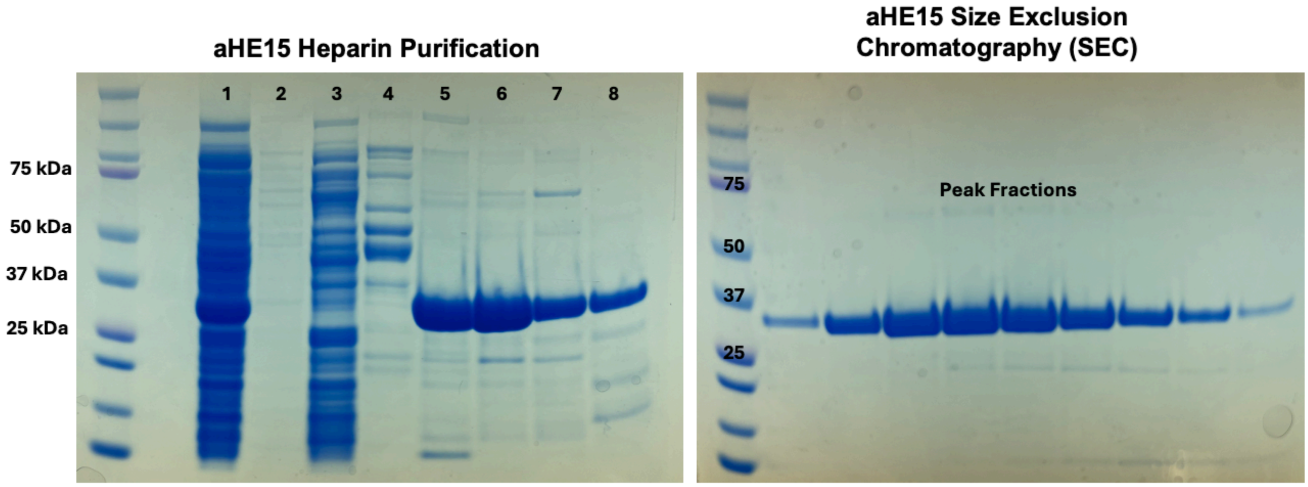

Lane 1 Supernatant

Lane 2 Flow-through

Lane 3 Wash

Lanes 4-8 Heparin gradient elution fractions

Supplementary Figure S6. Amino acid sequence alignment of the characterized I-OnuI clade enzymes with reconstructed ancestors aHE11 and aHE15 and purification of recombinant aHE15 protein. (A) Fully conserved positions are highlighted in cyan, with the conserved sidechains listed below the alignment. **(B)** Recombinant aHE15 protein was purified using a two-step protocol, including heparin purification followed by size exclusion chromatography (SEC). The contents of lanes on the gel from the heparin purification are listed below the gel, and the aHE15 protein is expected at a molecular weight of 33.5 kDa.

bioRxiv preprint doi: https://doi.org/10.64898/2026.07.13.738334; this version posted July 14, 2026. The copyright holder for this preprint (which was not certified by peer review) is the author/funder, who has granted bioRxiv a license to display the preprint in perpetuity. It is made available under a CC-BY 4.0 International license.

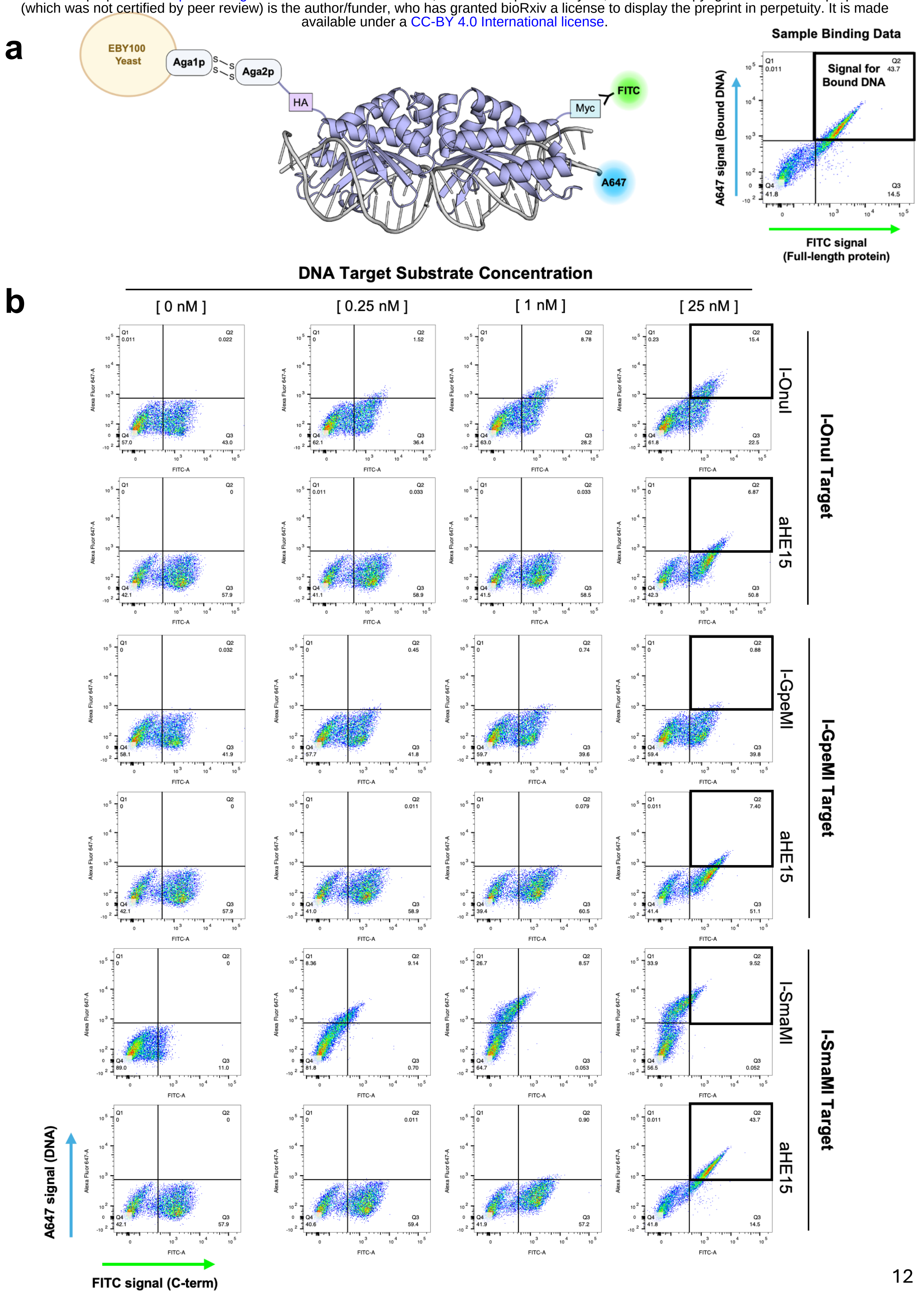

bioRxiv preprint doi: https://doi.org/10.64898/2026.07.13.738334; this version posted July 14, 2026. The copyright holder for this preprint (which was not certified by peer review) is the author/funder, who has granted bioRxiv a license to display the preprint in perpetuity. It is made available under a CC-BY 4.0 International license.

Supplementary Figure S7. Flow cytometric binding assay to assess the ability of ancestor aHE15 to bind the DNA target sequences of related modern-day enzymes. (A) Schematic of the flow cytometric DNA binding assay showing an example of strong binding. Surface-expressed LHE is stained with anti-Myc-FITC to track full-length protein, and a double-stranded A647-labeled DNA target substrate is provided free in solution (no tethering). Binding of the target substrate is indicated by the presence of A647 signal in the upper portion of flow plot with FITC signal (full length protein) plotted on the x-axis and A647 signal (on the substrate DNA) on the y-axis. A bold black box highlights the portion of the flow plot where evidence of binding can be observed. **(B)** Surface-expressed aHE15 was analyzed for binding of A647-labeled DNA substrates containing the target site sequences of I-Onu, I-GpeMI, and I-SmaMI and compared to the ability of each modern-day enzyme to bind its own target sequence. All surface-expressed LHEs were stained with anti-Myc-FITC to track the presence of full-length enzyme (FITC signal, x-axis), and incubated with A647-labeled DNA target substrate ranging in concentration from 0 nM up to 25 nM (A647 signal, y-axis).

bioRxiv preprint doi: https://doi.org/10.64898/2026.07.13.738334; this version posted July 14, 2026. The copyright holder for this preprint (which was not certified by peer review) is the author/funder, who has granted bioRxiv a license to display the preprint in perpetuity. It is made available under a CC-BY 4.0 International license.

a

SELEX0 Target Site Template

Forw Primer Binding Site	30x N Randomized Target	Rev Primer Binding Site

CAGGGATCCATGCACTGTACGTTTNNNNNNNNNNNNNNNNNNNNNNNNNNNNNNAAACCACTTGACTGCGGATCCT

SELEX forward primer	SELEX reverse primer
CAGGGATCCATGCACTGTACG	AGGATCCGCAGTCAAGTGG

b

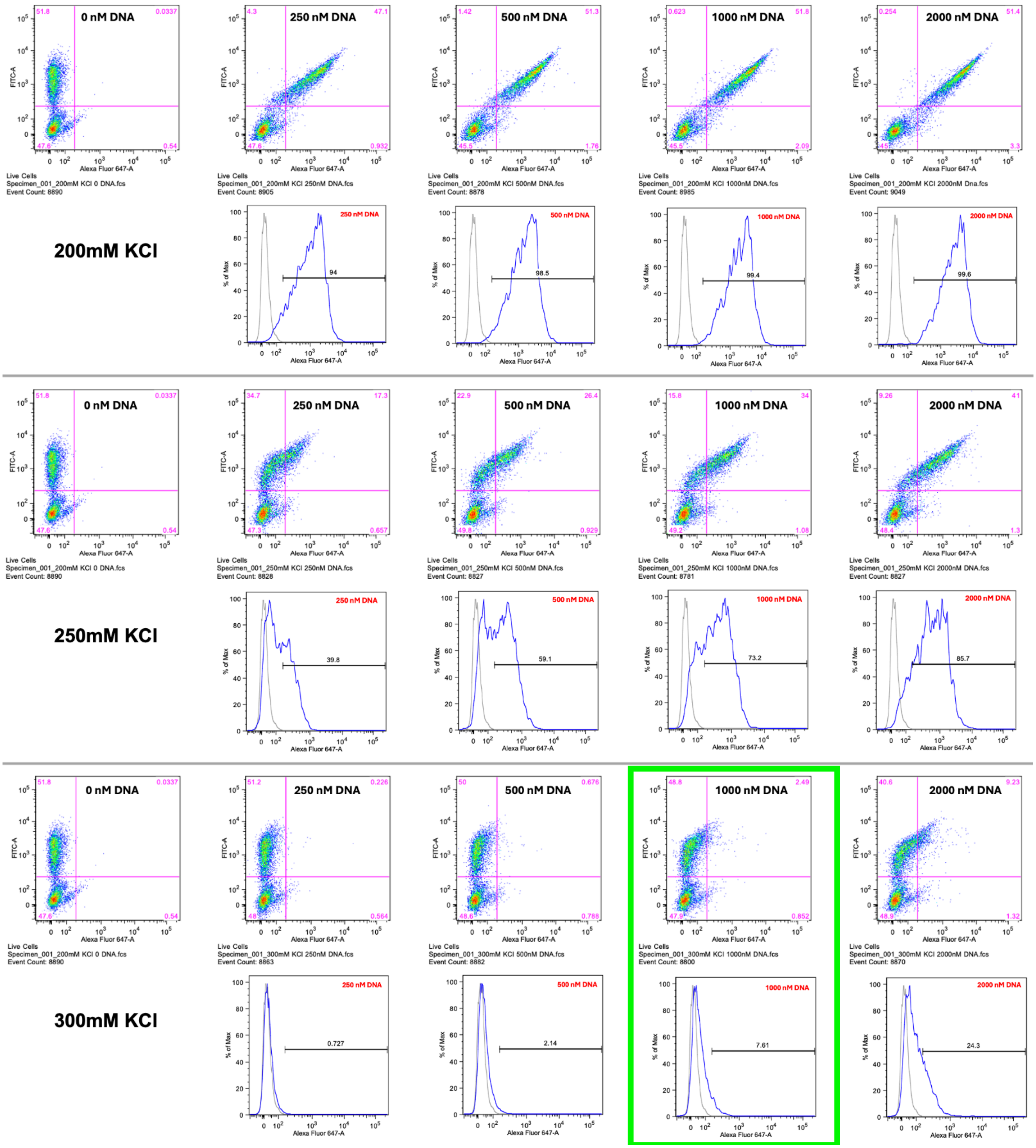

bioRxiv preprint doi: https://doi.org/10.64898/2026.07.13.738334; this version posted July 14, 2026. The copyright holder for this preprint (which was not certified by peer review) is the author/funder, who has granted bioRxiv a license to display the preprint in perpetuity. It is made available under a CC-BY 4.0 International license.

Supplementary Figure S8: DNA target substrate design and parameter optimization for the binding-based SELEX protocol. (A) The SELEX0 DNA target substrate library was generated using a single stranded template consisting of 30 basepairs of fully randomized DNA sequence (“30xN”) flanked by 24 bp forward and 22 bp reverse primer sequences. Three thymidine bases preceding and three adenine bases following the 30xN sequence are designed to discourage high-affinity binding to the constant regions of the substrate. Double-stranded DNA target substrate was generated by PCR amplification with forward and reverse SELEX primers. Fluorescently labeled DNA target substrate was generated using an A647-labeled reverse primer. **(B)** The concentration of KCl in the binding buffer and total DNA target substrate in the reaction both needed to be optimized to allow for approximately 5% overall binding of the SELEX0 target library. aHE15 was expressed on the surface of yeast and assayed for binding using an A647-labeled SELEX0 substrate. KCl in the binding buffer was assayed at 200 mM, 250 mM, and 300 mM concentrations in combination with total SELEX0 substrate concentrations ranging from 250 nM – 2000 nM. The final parameters selected for the SELEX experiment included 300 mM KCl and 1000 nM SELEX0 target site library substrate, which allowed for binding of approximately 7.6% of the SELEX0 library (bold green box).

bioRxiv preprint doi: https://doi.org/10.64898/2026.07.13.738334; this version posted July 14, 2026. The copyright holder for this preprint (which was not certified by peer review) is the author/funder, who has granted bioRxiv a license to display the preprint in perpetuity. It is made available under a CC-BY 4.0 International license.

SELEX0 Target Site Template

Constant Region — **30x N Randomized Target** — **Constant Region**

CAGGGATCCATGCACTGTACGTTTNNNNNNNNNNNNNNNNNNNNNNNNNNNNNN**AAACCACTTGACTGCGGATCCT**

Colony sequencing reads after Cleavage Selection Round 1 of 4:

CAGGGATCCATGCACTGTACGTTTAGCGCTTCGTTTATTGTGGTAAACTTCTAAAAACCACTTGACTGCGGATCCG
CAGGGATCCATGCACTGTACGTTTCTAGCGCACTTGTGGTCGAAATCAGTCTCTAAACCACTTGACTGCGGATCCT
CAGGGATCCATGCACTGTACGTTTTAGCGCTTTCACTGTTGGTAAAAAGTTTTCAAACCACTTGACTGCGGATCCG
CAGGGATCCATGCACTGTACGTTTAGCGCTGTGGCGACGTTGTGGTAAAAGTTTAAACCACTTGACTGCGGATCCT
CAGGGATCCATGCACTGTACGTTTAGCTCTCTTTTGTAGGTAAAGGTCATCTTGAAACCACTTGACTGCGGATCCT
CAGGGATCCATGCACTGTACGTTTAGCGCGATACAATGCTTTATCTTATGTGGTAAACCACTTGACTGCGGATCCT
CAGGGATCCATGCACTGTACGTTTTAGCATCGATGTTTGGGTAAAAGGGCTAAAAAACCACTTGACTGCGGATCCG
CAGGGATCCATGCACTGTACGTTTCAGTGGTATAGCTCGTGGGAGGTATAGCGTAAACCACTTGACTGCGGATCCT
CAGGGATCCATGCACTGTACGTTTGTGGTAATTTGATATTAAAATTAGCGCTCTAAACCACTTGACTGCGGATCCG
CAGGGATCCATGCACTGTACGTTTAGTTGTCATTACAGGTAAAGGGCCAATTA-AAACCACTTGACTGCGGATCCT
CAGGGATCCATGCACTGTACGTTTAGGGTTACCGTAAAGGTCGTGAATTTGATGAAACCACTTGACTGCGGATCCT
CAGGGATCCATGCACTGTACGTTTAGCATCTGTGGGGTAAACGGCTGTAACTAGAAACCACTTGACTGCGGATCCG
CAGGGATCCATGCACTGTACGTTTAGCTGCCTAGGTAAAGGGCCAAAATTATCTAAACCACTTGACTGCGGATCCT
CAGGGATCCATGCACTGTACGTTTTAGCGTTTCGGTAAAAAGCCACAAATCAACAAACCACTTGACTGCGGATCCT
CAGGGATCCATGCACTGTACGTTTAGCGCAGCCCGTGGTAAACAGTGGTGCTAAAAACCACTTGACTGCGGATCCT
CAGGGATCCATGCACTGTACGTTTAGCACGGTGGGGTAAAGGGCTTCTTCTAAGAAACCACTTGACTGCGGATCCG
CAGGGATCCATGCACTGTACGTTTTAGGGCTTTGTCCGTAAAAAGGCTTTAAAGAAACCACTTGACTGCGGATCCT
CAGGGATCCATGCACTGTACGTTTATAGCGTCCCCGGTATCTAAGTTTAGTGAGAAACCACTTGACTGCGGATCCT
CAGGGATCCATGCACTGTACGTTTAACCGGTTAGGTCTCAGGAGTAAGCGAATTAAACCACTTGACTGCGGATCCT
CAGGGATCCATGCACTGTACGTTTAGCTGAGTTGCTGGTAAAGGCAGAGTGTAAAAACCACTTGACTGCGGATCCT
CAGGGATCCATGCACTGTACGTTTAGCGCCTCATCGTGGTAAATTGTACTTTCTAAACCACTTGACTGCGGATCCG
CAGGGATCCATGCACTGTACGTTTAAGGTGCTTCGCCATAAAGGGGGGTTTATGAAACCACTTGACTGCGGATCCT
CAGGGATCCATGCACTGTACGTTTAGTGCCACATATTTAGCAGTAAAGGGCTAGAAACCACTTGACTGCGGATCCT
CAGGGATCCATGCACTGTACGTTTAGCGACTTGTTTAATGAzTGGTAAAGGGCAAAAACCACTTGACTGCGGATCCT
CAGGGATCCATGCACTGTACGTTTTTATAGGACATTTAGGGGTAAAAAAAGGGCAAACCACTTGACTGCGGATCCT
CAGGGATCCATGCACTGTACGTTTAGGTATAGCATTCTGAGGTATAGCTTTCACAAACCACTTGACTGCGGATCCT
CAGGGATCCATGCACTGTACGTTTAATAGCGTCTCGGTCTTGATGTTCAGAGTAAAACCACTTGACTGCGGATCCG
CAGGGATCCATGCACTGTACGTTTGCTTATTAGCGCCTGTTGTGGTAATATGTCAAACCACTTGACTGCGGATCCT
CAGGGATCCATGCACTGTACGTTTAGCGCGATTGTGGTAAATAATTACATCCTTAAACCACTTGACTGCGGATCCT
CAGGGATCCATGCACTGTACGTTTGTGGTATCTTAAATAGCGCTGGTTACTTTAAAACCACTTGACTGCGGATCCT
CAGGGATCCATGCACTGTACGTTTCATAGGGGTATAGCTACTTCAGGTATAGAGAAACCACTTGACTGCGGATCCT
CAGGGATCCATGCACTGTACGTTTAAAGCAGTCTCGGTTCAAAGGGCAAGCAAAAAACCACTTGACTGCGGATCCG
CAGGGATCCATGCACTGTACGTTTAGCGCCAAAGGGGTAAAGGGCTACGAATTTAAACCACTTGACTGCGGATCCT
CAGGGATCCATGCACTGTACGTTTTAGTGCGTGAAGTAAAAGGGCTTAATGTGAAAACCACTTGACTGCGGATCCG
CAGGGATCCATGCACTGTACGTTTTTAGCGCTATCGTGGTAAAGGCGTGTTTAGAAACCACTTGACTGCGGATCCT
CAGGGATCCATGCACTGTACGTTTAAGCGCTCTGTGGTTAATATTGTCAGATACAAACCACTTGACTGCGGATCCG
CAGGGATCCATGCACTGTACGTTTTAGAGCATTGGGGTAAAAGGTTTATAGATTAAACCACTTGACTGCGGATCCT
CAGGGATCCATGCACTGTACGTTTGATAATAGGGCCAATGAAAGTATTATTTGAAACCACTTGACTGCGGATCCT
CAGGGATCCATGCACTGTACGTTTAGGGCGCTGTAGTAAACGGTTACTTGCTTCAAACCACTTGACTGCGGATCCT

Colony sequencing reads after Cleavage Selection Round 2 of 4:

CAGGGATCCATGCACTGTACGTTTAGCAATTTCTTGGTAAAGGGCTTTGCCACTAAACCACTTGACTGCGGATCCG
CAGGGATCCATGCACTGTACGTTT**TAGGGATGTGTGTACGTAAAAGGGCAACTT**AAACCACTTGACTGCGGATCCT
CAGGGATCCATGCACTGTACGTTTAGTGCTTTTTGGTAGTAAAGGGCGCATCTAAAACCACTTGACTGCGGATCCT
CAGGGATCCATGCACTGTACGTTTTCGTAGAGCTCCCACTAAAAATAGTGAATCAAACCACTTGACTGCGGATCCT
CAGGGATCCATGCACTGTACGTTTTTAGCGCTTATGTGGTAAATTTACGCCACGAAACCACTTGACTGCGGATCCT
CAGGGATCCATGCACTGTACGTTTTAATTTGGTAAGCGCTGTCGCCGGTTAGAGAAACCACTTGACTGCGGATCCT
CAGGGATCCATGCACTGTACGTTTAACTTAGCGCTTTGTGGTAAGGTTTGATCAAAACCACTTGACTGCGGATCCT
CAGGGATCCATGCACTGTACGTTTACTTAGAGCTCGCTGTTTAATTATCATTTGAAACCACTTGACTGCGGATCCT
CAGGGATCCATGCACTGTACGTTTTTAGCGCCATTGTGGTAAAGTACAGTTTTAAAACCACTTGACTGCGGATCCT
CAGGGATCCATGCACTGTACGTTTGGTTAGCAGCCCCGGTAAGCGGGTTTTAATAAACCACTTGACTGCGGATCCT
CAGGGATCCATGCACTGTACGTTTCGTGGTAAGCGGCGTTTAGGTTAGCAAAAGAAACCACTTGACTGCGGATCCG
CAGGGATCCATGCACTGTACGGTTTACAGTCTCCTTTTTAGGGCGAACGGCAGTAAAACCACTTGACTGCGGATCCG
CAGGGATCCATGCACTGTACGTTTATTGATTGTGGTAACACGGTTAGCGCT--TAAAACCACTTGACTGCGGTC-T
CAGGGATCCATGCACTGTACGTTTAGCGCTCTTGTGGTGTAACGAATAGTTGTAAAACCACTTGACTGCGGATCCT
CAGGGATCCATGCACTGTACGTTT**TAGGGATGTGTGTACGTAAAAGGGCAACTT**AAACCACTTGACTGCGGATCCT
CAGGGATCCATGCACTGTACGTTTTCATTTAGCTGGACCGCTGGTAAATGATTTAAACCACTTGACTGCGGATCCT
CAGGGATCCATGCACTGTACGTTTTGACGTAGAGCTCCCCTAAAATATATTCTCAAACCACTTGACTGCGGATCCG
CAGGGATCCATGCACTGTACGTTTATTGAATTGTCTATAAAGGGCTAGTGAACTAAACCACTTGACTGCGGATCCT
CAGGGATCCATGCACTGTACGTTTTTTAGCGCACGTGGTATATAACGTCTATTCAAACCACTTGACTGCGGATCCT
CAGGGATCCATGCACTGTACGTTTAGCTCCTTGTGGTGAGGTAAAAGGCCCTGCAAACCACTTGACTGCGGATCCT
CAGGGATCCATGCACTGTACGTTTACGTAGAGCTCCCAACAATTTGAGTGTTTTAAACCACTTGACTGCGGATCCT
CAGGGATCCATGCACTGTACGTTTTAGTTTCGCAAGTAAAAGGGCTATAGCTTAAAACCACTTGACTGCGGATCCG
CAGGGATCCATGCACTGTACGTTTGGTCATATTAGGGCGTGTGAAAGTAATATGAAACCACTTGACTGCGGATCCT
CAGGGATCCATGCACTGTACGTTTAGCGCATCGCCTGGTAAAAGTTGTGTTTTAAAACCACTTGACTGCGGATCCT
CAGGGATCCATGCACTGTACGTTTAGGGCCCGAAGTAAAGGTCGAGTGTAATTCAAAACCACTTGACTGCGGATCCT
CAGGGATCCATGCACTGTACGTTTGGTAAGCTCTTGCTGGTTAGCTGGCGTTAAAAACCACTTGACTGCGGATCCT
CAGGGATCCATGCACTGTACGTTTGTGGTATTTCATAGCGCCTGAATTTTCTATAAACCACTTGACTGCGGATCCG
CAGGGATCCATGCACTGTACGTTTTAGATAGCTCCACGGTTGAGGTAAAAGGGCAAACCACTTGACTGCGGATCCT
CAGGGATCCATGCACTGTACGTTTAGCGCCTACGTGGTAAAGGTTGTCGTTTTTAAACCACTTGACTGCGGATCCT
CAGGGATCCATGCACTGTACGTTTAGCGCCCTGCTTGTGGTAAATGTCGGCTAGAAACCACTTGACTGCGGATCCT
CAGGGATCCATGCACTGTACGTTTTAGCGCTGTTGTGGTACTAGTTCAGTTTAAAAACCACTTGACTGCGGATCCG
CAGGGATCCATGCACTGTACGTTTAGCGCTATGGTGGTATAACTTATCAACCC-AAACCACTTGACTGCGGATCCG
CAGGGATCCATGCACTGTACGTTTTAGCATAGTGATGGTAAAAGGTCTTGTCATAAAACACTTGACTGCGGATCCT
CAGGGATCCATGCACTGTACGTTTGTGGTAAAAGGCTTATTTTAGCGCTCATAGAAACCACTTGACTGCGGATCCT
CAGGGATCCATGCACTGTACGTTTAAAGTTCTCACCCCTTTAAAGGGCTTGAATAAACCACTTGACTGCGGATCCG
CAGGGATCCATGCACTGTACGTTTTAGCGCCTACGTTGGTAAATAGCGTTAGAGAAACCACTTGACTGCGGATCCG
CAGGGATCCATGCACTGTACGTTTAGTGCTTTCGCAGTAAAAGGTCGGTCGTCAAAACCACTTGACTGCGGATCCT
CAGGGATCCATGCACTGTACGTTTTTAGGGTCATACACTAAAAAAGTCGGATAGAAACCACTTGACTGCGGATCCT
CAGGGATCCATGCACTGTACGTTTTCAGACGGTAAGCATCACGAGGTTAGCGGAAAACCACTTGACTGCGGATCCT
CAGGGATCCATGCACTGTACGTTTAGCGTTTATAGGTAAAGGGCACATGGGCTTAAACCACTTGACTGCGGATCCT
CAGGGATCCATGCACTGTACGTTTGCTGTGGTAAGAGTCTTAGCGCCCTTTCTAAAACCACTTGACTGCGGATCCT

Colony sequencing reads after Cleavage Selection Round 3 of 4:

CAGGGATCCATGCACTGTACGTTTGGTAAGCGCTAGTTCGGTTAGTCGGGTTAGAAACCACTTGACTGCGGATCCT
CAGGGATCCATGCACTGTACGTTTGTGGTATATTTCCAATATAGCGCCTCAATCAAACCACTTGACTGCGGATCCT
CAGGGATCCATGCACTGTACGTTTAGGGCCTTGTTCGTAAAAGGCTTTGATCATAAACCACTTGACTGCGGATCCT
CAGGGATCCATGCACTGTACGTTTTAGCGCTATCGTGGTAAATTAGTTATAACCAAACCACTTGACTGCGGATCCT
CAGGGATCCATGCACTGTACGTTTCGCGGTATAGCACCACGGGGTATAGCACATAAACCACTTGACTGCGGATCCG
CAGGGATCCATGCACTGTACGTTTAGTGTTCTCCGGTAAAAGGCTTAGACTGTAAAACCACTTGACTGCGGATCCT
CAGGGATCCATGCACTGTACGTTTTAGCGCTTTATTGTTGGTAAAAGAATATGGAAACCACTTGACTGCGGATCCT
CAGGGATCCATGCACTGTACGTTTAGTGCTATGGCGGTAAAAGGCAATAGTCAGAAACCACTTGACTGCGGATCCT
CAGGGATCCATGCACTGTACGTTTAGCGTACTCGGTAAAAGGCCGGGCCAAAGCAAACCACTTGACTGCGGATCCG
CAGGGATCCATGCACTGTACGTTTTAGCTGGTTGGCTGGTAAAAGGTCATTCTTAAACCACTTGACTGCGGATCCT
CAGGGATCCATGCTCTGTACGTTTGGTAAGGGCTTTCTTGCAGTTAGCGAAGTAAAACCACTTGACTGCGGATCCT
CAGGGATCCATGCACTGTACGTTT**GAGTCGGTATACATAGGGTTGTATAGCGGT**AAACCACTTGACTGCGGATCCG
CAGGGATCCATGCACTGTACGTTTAATAGCGCTATGTGGTCTTTCGGGTAGGCAAAACCACTTGACTGCGGATCCT
CAGGGATCCATGCACTGTACGTTTCTGGTTAGTGTTAGCGCTTTGTGGTAAGTTAAACCACTTGACTGCGGATCCT
CAGGGATCCATGCACTGTACGTTTAGCGGGGCACCCGTTTTAATCGGGTTTGGTAAACCACTTGACTGCGGATCCT
CAGGGATCCATGCACTGTACGTTTAGCGCTGAGCGGTAAAGGGCTACGTCACAAAAACCACTTGACTGCGGATCCT
CAGGGATCCATGCACTGTACGTTTTGTGGTAAAATTTATAGGTTGGATTGTGGTAAACCACTTGACTGCGGATCCG
CAGGGATCCATGCACTGTACGTTTAGCTGGTACTCGGTAAAAGGCTTTCATTCGAAACCACTTGACTGCGGATCCG
CAGGGATCCATGCACTGTACGTTTTAGCGCTTTTTGTTGGTAAAACTTTAGAACAAACCACTTGACTGCGGATCCG
CAGGGATCCATGCACTGTACGTTT**TAGGGATGTGTGTACGTAAAAGGGCAACTT**AAACCACTTGACTGCGGATCCT
CAGGGATCCATGCACTGTACGTTTAGCGCGCTGTGGTAAATAGTAGCGTTACTTAAACCACTTGACTGCGGATCCT
CAGGGATCCATGCACTGTACGTTTGTTGAGGTGAACTTCGTTTAACGCCAATCTAAACCACTTGACTGCGGATCCT
CAGGGATCCATGCACTGTACGTTTACGTAGAGCTCCCTCTCGGCGATTCTACTAAAACCACTTGACTGCGGATCCT
CAGGGATCCATGCACTGTACGTTTGGAGGTAAAGTCCTTTGGGTTTAGCTAAGAAAACCACTTGACTGCGGATCCT
CAGGGATCCATGCACTGTACGTTTAGCTATACCTCTATGGTAAAGGGCTTTAAAAAACCACTTGACTGCGGATCCT
CAGGGATCCATGCACTGTACGTTT**GAGTCGGTATACATAGGGTTGTATAGCGGT**AAACCACTTGACTGCGGATCCG
CAGGGATCCATGCACTGTACGTTT**GAGTCGGTATACATAGGGTTGTATAGCGGT**AAACCACTTGACTGCGGATCCG
CAGGGATCCATGCACTGTACGTTTCGCCGAAAATAGAGCTCCCTTCGACCATTTAAACCACTTGACTGCGGATCCT
CAGGGATCCATGCACTGTACGTTTAGCACTCTGCCATGGTAAAGGGCCTTACAGAAACCACTTGACTGCGGATCCT
CAGGGATCCATGCACTGTACGTTTAGATTTAGCGCCATCTTGTGGTAAATCGTGAAACCACTTGACTGCGGATCCT
CAGGGATCCATGCACTGTACGTTTAGCGTTTAGTCGGTAAAGGGCACCTACGAGAAACCACTTGACTGCGGATCCG
CAGGGATCCATGCACTGTACGTTTAGGCTCTAATAGCGGTATGTCGGTATTATAAAACCACTTGACTGCGGATCCT
CAGGGATCCATGCACTGTACGTTTATTGCTCGCAAATAAAGGGCTCCTTGTCTTAAACCACTTGACTGCGGATCCT
CAGGGATCCATGCACTGTACGTTTTAAGGCCACGCACATAAAAGGGCTATTCTAAAACCACTTGACTGCGGATCCG
CAGGGATCCATGCACTGTACGTTTTGCAGTTATAGGGTTACAGTATAACGCCTTAAACCACTTGACTGCGGATCCT

Colony sequencing reads after Cleavage Selection Round 4 of 4:

CAGGGATCCATGCACTGTACGAAT**GCGGTATAAGCACCCCTATAGCGCTATTTA**AAACCACTTGACTGCGGATCCT
CAGGGATCCATGCACTGTACGTTT**TAGGGATGTGTGTACGTAAAAGGGCAACTT**AAACCACTTGACTGCGGATCCT
CAGGGATCCATGCACTGTACGTTT**GAGTCGGTATACATAGGGTTGTATAGCGGT**AAACCACTTGACTGCGGATCCG
CAGGGATCCATGCACTGTACGTTT**GAGTCGGTATACATAGGGTTGTATAGCGGT**AAACCACTTGACTGCGGATCCG
CAGGGATCCATGCACTGTACGTTT**GCGGTATAAGCACCCCTATAGCGCTATTTA**AAACCACTTGACTGCGGATCCT
CAGGGATCCATGCACTGTACGTTT**TAGGGATGTGTGTACGTAAAAGGGCAACTT**AAACCACTTGACTGCGGATCCT
CAGGGATCCATGCACTGTACGTTT**GAGTCGGTATACATAGGGTTGTATAGCGGT**AAACCACTTGACTGCGGATCCG
CAGGGATCCATGCACTGTACGTTT**GAGTCGGTATACATAGGGTTGTATAGCGGT**AAACCACTTGACTGCGGATCCG
CAGGGATCCATGCACTGTACGTTTAGTTCGGTAATAGCGCTATCGTGGTATTTTAAACCACTTGACTGCGGATCCG
CAGGGATCCATGCACTGTACGTTT**GAGTCGGTATACATAGGGTTGTATAGCGGT**AAACCACTTGACTGCGGATCCG
CAGGGATCCATGCACTGTACGTTT**GAGTCGGTATACATAGGGTTGTATAGCGGT**AAACCACTTGACTGCGGATCCG
CAGGGATCCATGCACTGTACGTTT**GAGTCGGTATACATAGGGTTGTATAGCGGT**AAACCACTTGACTGCGGATCCG
CAGGGATCCATGCACTGTACGTTT**TAGGGATGTGTGTACGTAAAAGGGCAACTT**AAACCACTTGACTGCGGATCCT
CAGGGATCCATGCACTGTACGTTTTAGCGCCTCCGTGGTCAAATTAAATTGTGGAAACCACTTGACTGCGGATCCG
CAGGGATCCATGCACTGTACGTTT**GAGTCGGTATACATAGGGTTGTATAGCGGT**AAACCACTTGACTGCGGATCCG
CAGGGATCCATGCACTGTACGTTT**TAGGGATGTGTGTACGTAAAAGGGCAACTT**AAACCACTTGACTGCGGATCCT
CAGGGATCCATGCACTGTACGTTT**GAGTCGGTATACATAGGGTTGTATAGCGGT**AAACCACTTGACTGCGGATCCG
CAGGGATCCATGCACTGTACGTTT**GAGTCGGTATACATAGGGTTGTATAGCGGT**AAACCACTTGACTGCGGATCCG
CAGGGATCCATGCACTGTACGTTT**GAGTCGGTATACATAGGGTTGTATAGCGGT**AAACCACTTGACTGCGGATCCG
CAGGGATCCATGCACTGTACGTTT**GAGTCGGTATACATAGGGTTGTATAGCGGT**AAACCACTTGACTGCGGATCCG
CAGGGATCCATGCACTGTACGTTT**GAGTCGGTATACATAGGGTTGTATAGCGGT**AAACCACTTGACTGCGGATCCG
CAGGGATCCATGCACTGTACGTTTAGCGATTGACGGTAAAGGTTTCATATATTCAAACCACTTGACTGCGGATCCT
CAGGGATCCATGCACTGTACGTTT**GAGTCGGTATACATAGGGTTGTATAGCGGT**AAACCACTTGACTGCGGATCCG
CAGGGATCCATGCACTGTACGTTTAGCAATTCTTGTATGGTAAAGGGCTGATATAAACCACTTGACTGCGGATCCT
CAGGGATCCATGCACTGTACGTTTAGCAGTTGTAAGGTTGGTAAAGGGCGTCTTAAACCACTTGACTGCGGATCCT
CAGGGATCCATGCACTGTACGTTT**GAGTCGGTATACATAGGGTTGTATAGCGGT**AAACCACTTGACTGCGGATCCG
CAGGGATCCATGCACTGTACGTTTTAAACCTCCGACCGTTTAAAAGGTCTAATGAAACCACTTGACTGCGGATCCT
CAGGGATCCATGCACTGTACGTTT**GAGTCGGTATACATAGGGTTGTATAGCGGT**AAACCACTTGACTGCGGATCCG
CAGGGATCCATGCACTGTACGTTT**GAGTCGGTATACATAGGGTTGTATAGCGGT**AAACCACTTGACTGCGGATCCG

17xSeq Enriched Target Site Sequence **GAGTCGGTATACATAGGGTTGTATAGCGGT**

4xSeq Enriched Target Site Sequence **TAGGGATGTGTGTACGTAAAAGGGCAACTT**

2xSeq Enriched Target Site Sequence **GCGGTATAAGCACCCCTATAGCGCTATTTA**

bioRxiv preprint doi: https://doi.org/10.64898/2026.07.13.738334; this version posted July 14, 2026. The copyright holder for this preprint (which was not certified by peer review) is the author/funder, who has granted bioRxiv a license to display the preprint in perpetuity. It is made available under a CC-BY 4.0 International license.

Supplementary Figure S9. Sequencing results from 4 rounds of cleavage selection. 48 colonies were sequenced after each round of the cleavage selection to look for enrichment of sequences cleavable by the aHE15 enzyme. Failed or ambiguous sequence reads are not listed here. The constant regions of the DNA target substrate mark the boundaries of the 30 bp target sequence under analysis and are colored with blue text. After four rounds of cleavage (bottom right), there were three different target sequences that were enriched to some degree, with the "2xSeq" target present two times (purple bold text), the "4xSeq" target present four times (black bold text), and the "17xSeq" target present in 17 of the 29 good sequence reads (red bold text). The 17xSeq target sequence was also present 3 times in the colonies sequenced after Round 3, and the 4xSeq target was present once after Round 3 and two times after Round 2.

bioRxiv preprint doi: https://doi.org/10.64898/2026.07.13.738334; this version posted July 14, 2026. The copyright holder for this preprint (which was not certified by peer review) is the author/funder, who has granted bioRxiv a license to display the preprint in perpetuity. It is made available under a CC-BY 4.0 International license.

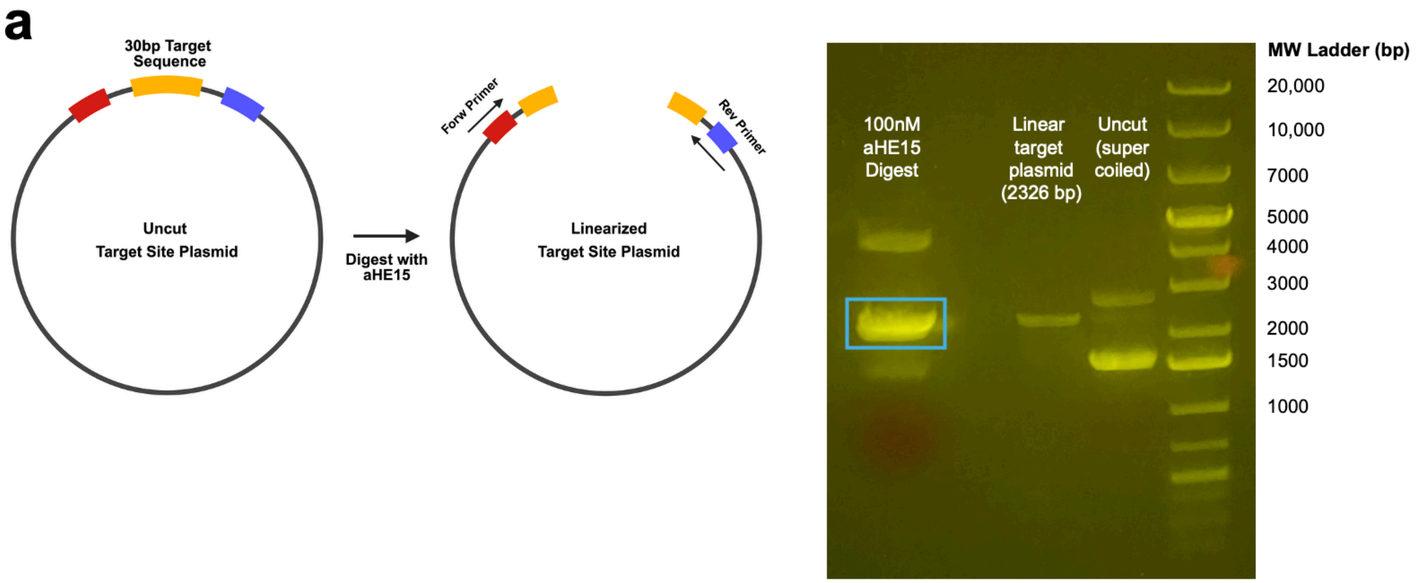

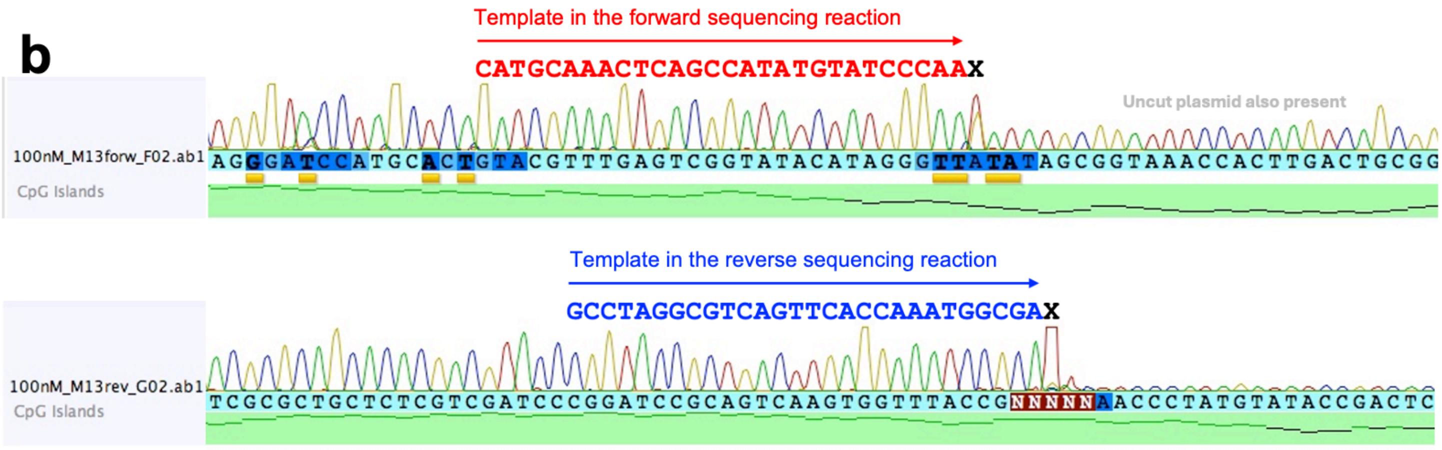

c

Double-stranded 30xN SELEX0 library substrate:

Primer Binding Sequence   Primer Binding Sequence

CAGGGATCCATGCACTGTACGTTTNNNNNNNNNNNNNNNNNNNNNNNNNNNNNNAAACCACTTGACTGCGGATCCG
GTCCCTAGGTACGTGACATGCAAANNNNNNNNNNNNNNNNNNNNNNNNNNNNNNTTTGGTGAACTGACGCCTAGGC

Double-stranded 17xSeq target site:

Cut

CAGGGATCCATGCACTGTACGTTTGAGTCGGTATACATAGGGTTGTAT   AGCGGTAAACCACTTGACTGCGGATCCG
GTCCCTAGGTACGTGACATGCAAACTCAGCCATATGTATCCCAA   CATATCGCCATTTGGTGAACTGACGCCTAGGC

Cut

17xSeq 22bp target site with Central Four Highlighted:

CAGGGATCCATGCACTGTACGTTTGAGTCGGTATACATAGGGTTGTATAGCGGTAAACCACTTGACTGCGGATCCG
GTCCCTAGGTACGTGACATGCAAACTCAGCCATATGTATCCCAACATATCGCCATTTGGTGAACTGACGCCTAGGC

bioRxiv preprint doi: https://doi.org/10.64898/2026.07.13.738334; this version posted July 14, 2026. The copyright holder for this preprint (which was not certified by peer review) is the author/funder, who has granted bioRxiv a license to display the preprint in perpetuity. It is made available under a CC-BY 4.0 International license.

Supplementary Figure S10. Run-off sequencing to determine the center of the 17xSeq cleavable target sequence for aHE15. (A) Schematic representation of the run-off sequencing experiment. The 17xSeq target site plasmid contains the 30 bp target sequence at a position that could be sequenced by both forward and reverse sequencing primers. Recombinant purified aHE15 protein was used to digest the intact 17xSeq target site plasmid, and the linearized product (blue box in the gel) was extracted from an agarose gel and purified. A PvuI-linearized plasmid control was run alongside uncut/supercoiled plasmid in the gel to help determine the correct location of the linearized product for extraction. **(B)** Both forward and reverse primers were used to sequence the purified linear product in two separate Sanger sequencing reactions. The Taq polymerase in the BigDye sequencing master mix (Thermo Fisher Scientific) produced a large adenine peak in the sequencing chromatogram when it reached the end of the cleaved DNA template strand. The location of this large A peak was used to identify the precise location where aHE15 cleaved each strand of the DNA target. The sequencing chromatogram readout represents the complement of the actual template in the sequencing reaction, so the complementary sequence of the target site plasmid template in the reaction is listed above the chromatogram for each read. The sequence of the template in the forward sequencing reaction is listed in red text and the template in the reverse sequencing reaction is listed in blue text. A black **X** indicates the position in the DNA sequence of the cut made by the aHE15 enzyme, as indicated by the large “A” peak in the chromatogram. **(C)** The double-stranded sequence of the 30xN SELEX0 library target site substrate is listed for reference, followed by the double-stranded 17xSeq target sequence identified after round 4 of the cleavage selection. The locations of the observed cuts on each strand of the target sequence (identified by the two run-off sequencing reactions shown above) are marked with vertical red and blue lines, and spaces are introduced at the position of each cut to show the 4-base, 3’ overhangs generated by the aHE15 enzyme. Finally, the 22bp centered 17xSeq target is outlined with a rectangular box.

bioRxiv preprint doi: https://doi.org/10.64898/2026.07.13.738334; this version posted July 14, 2026. The copyright holder for this preprint (which was not certified by peer review) is the author/funder, who has granted bioRxiv a license to display the preprint in perpetuity. It is made available under a CC-BY 4.0 International license.

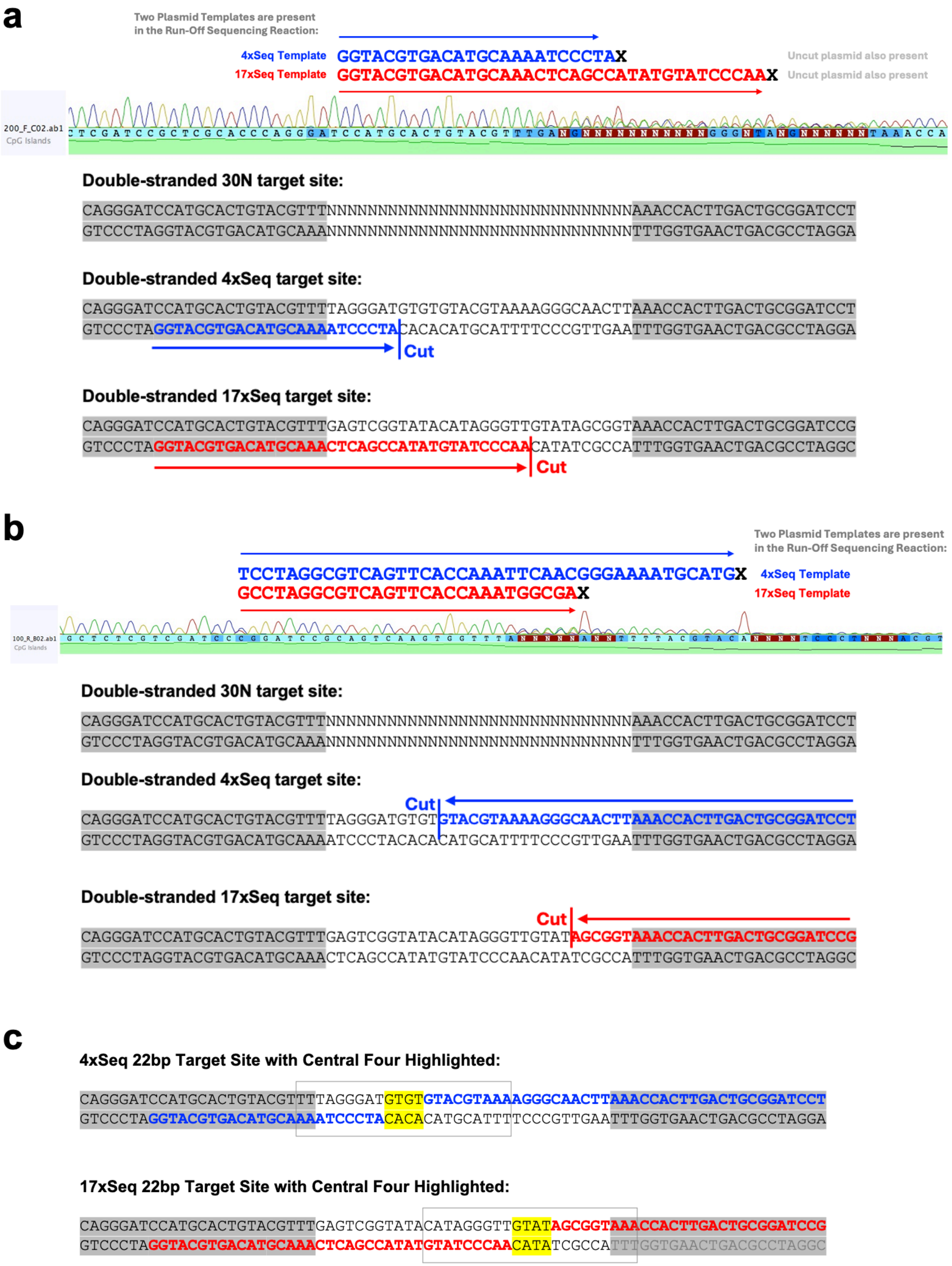

bioRxiv preprint doi: https://doi.org/10.64898/2026.07.13.738334; this version posted July 14, 2026. The copyright holder for this preprint (which was not certified by peer review) is the author/funder, who has granted bioRxiv a license to display the preprint in perpetuity. It is made available under a CC-BY 4.0 International license.

Supplementary Figure S11. Run-off sequencing to determine the center of the 4xSeq cleavable target sequence for aHE15. (A) A forward run-off sequencing reaction with linearized 4xSeq target site plasmid as the template revealed that the prep actually contained two different plasmids containing the target site plasmids for both the 4xSeq and 17xSeq sequences. It was possible to identify the chromatogram peaks corresponding to each of the two separate target sequences (4xSeq target in blue text and 17xSeq target in red text), as well as two separate large “A” peaks to represent two cleavage locations. There was also signal for uncut plasmid leftover in the digest reaction, as indicated by the presence of additional smaller peaks in the sequencing chromatogram after the location of cleavage. **(B)** The reverse run-off sequencing reaction also contained chromatograms for both the 4xSeq (blue text) and 17xSeq (red text) targets, as well as two separate large “A” peaks to designate the location of cleavage. **(C)** The run-off sequencing reactions allowed for the identification of the centered 22 bp target sequences for both the 4xSeq (blue) and 17xSeq (red) target sites, as outlined by rectangular boxes. The centered 22 bp 4xSeq target includes two of the flanking thymidines from the constant region of the SELEX target site template, and the centered 22bp 17x Seq target includes all three of the flanking adenines from the constant region. The center of the 17xSeq target site identified here matched and confirmed the data presented in **Supplementary Figure S10**.

bioRxiv preprint doi: https://doi.org/10.64898/2026.07.13.738334; this version posted July 14, 2026. The copyright holder for this preprint (which was not certified by peer review) is the author/funder, who has granted bioRxiv a license to display the preprint in perpetuity. It is made available under a CC-BY 4.0 International license.

```
I-OnuI Target    TTTCCACTTATTCAACCTTTTA
I-SmaMI Target   TATCCTCCATTATCAGGTGTAC
4x seq           TTTACGTACACACATCCCTAAA
17x seq          TTTACCGCTATACAACCCTATG
```

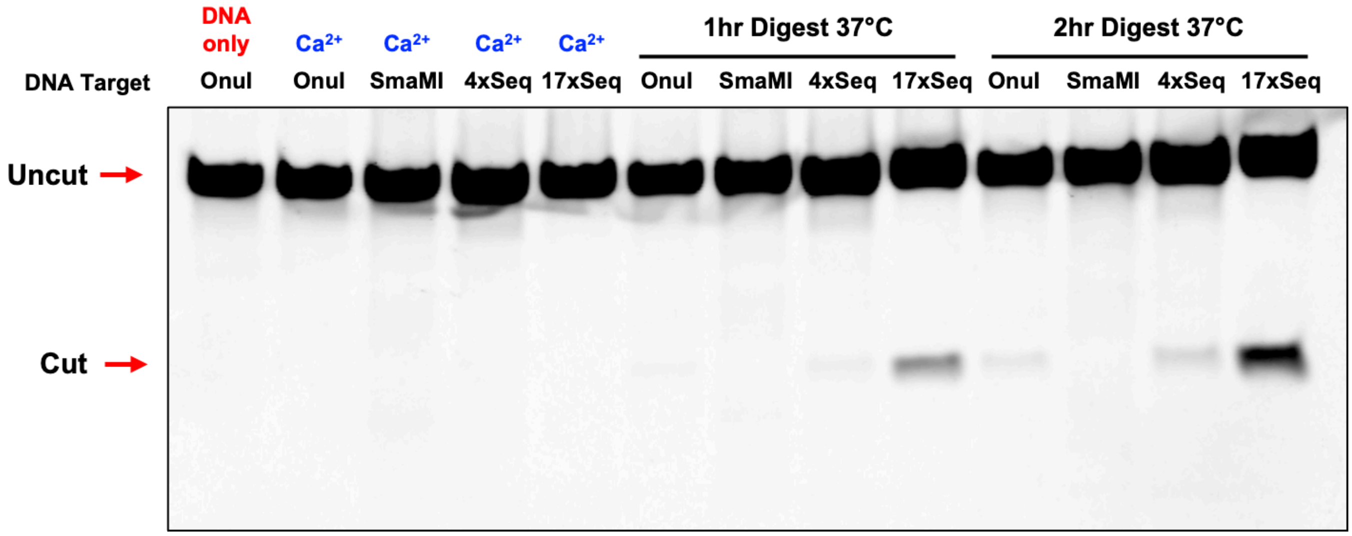

Supplementary Figure S12. Cleavage activity of aHE15 vs. the two most highly enriched sequences from the cleavage selection screen. Purified recombinant aHE15 was used to digest A647-labeled DNA substrates containing four different target sequences: I-OnuI, I-SmaMI, and both the 4xSeq and 17xSeq targets that were enriched after four rounds of the cleavage selection screen. The 22bp sequences for the 17xSeq and 4xSeq targets are shown as the reverse complement of the sequences presented in Figures S9 - S11, as this orientation lines up best with the verified I-OnuI target sequence. The sequences of the 17xSeq and 4xSeq targets are identical at 14 out of 22 positions. Cleavage products were separated by electrophoresis on an acrylamide gel and visualized by the presence of the A647 tag using a Typhoon fluorescence imager. Controls included a DNA-only lane (no enzyme) and digests performed in the presence of $CaCl_2$ (Ca^{2+} lanes) to indicate the position of uncut DNA substrate. The locations of cut and uncut DNA substrates are indicated on the gel with red arrows.

bioRxiv preprint doi: https://doi.org/10.64898/2026.07.13.738334; this version posted July 14, 2026. The copyright holder for this preprint (which was not certified by peer review) is the author/funder, who has granted bioRxiv a license to display the preprint in perpetuity. It is made available under a CC-BY 4.0 International license.

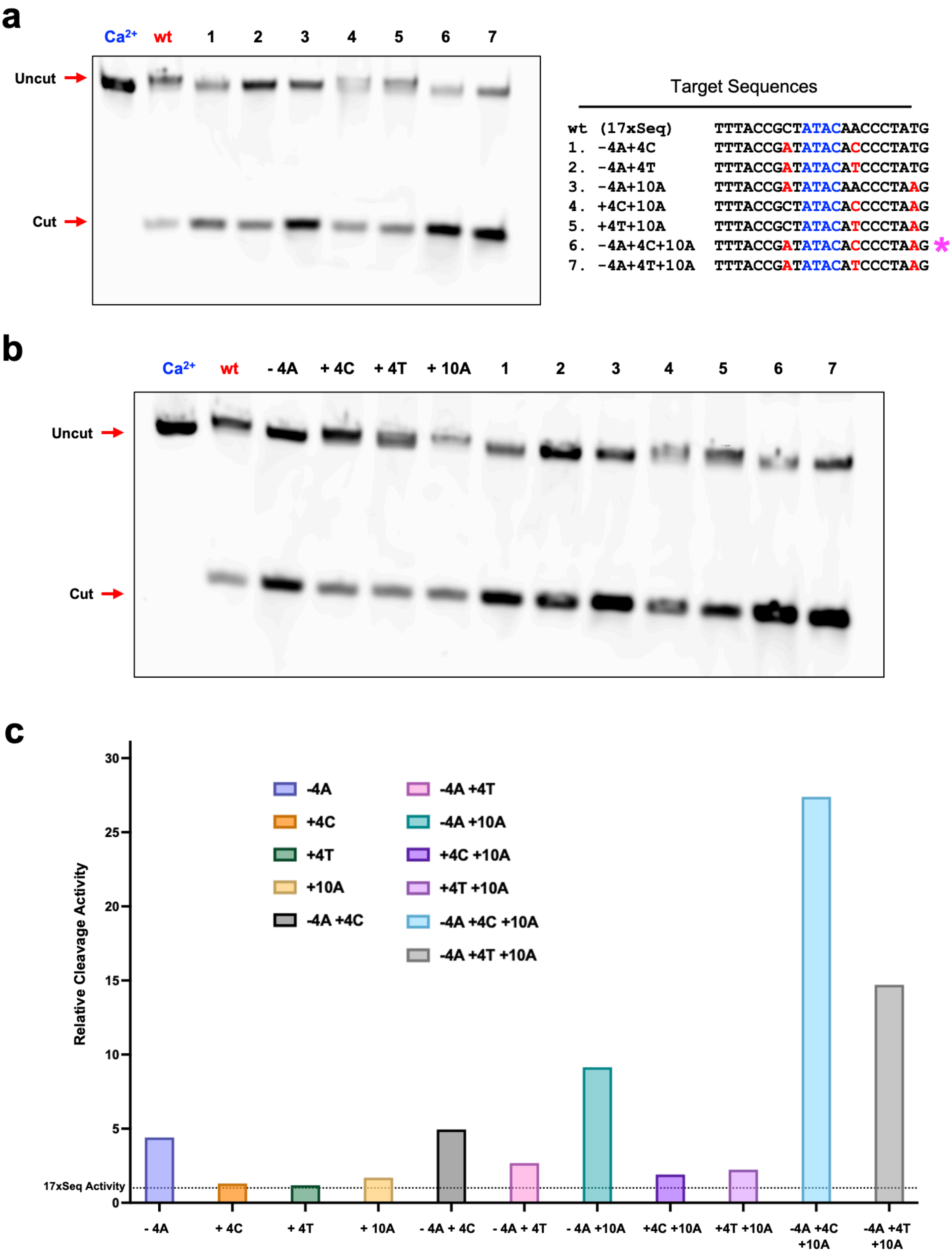

bioRxiv preprint doi: https://doi.org/10.64898/2026.07.13.738334; this version posted July 14, 2026. The copyright holder for this preprint (which was not certified by peer review) is the author/funder, who has granted bioRxiv a license to display the preprint in perpetuity. It is made available under a CC-BY 4.0 International license.

Supplementary Figure S13. Gel of in vitro digests used to identify an optimized alternative target sequence for aHE15. (A) Image of the acrylamide gel with cleavage products from *in vitro* digests of the seven combinatorial alternative target site sequences. The far-left lane contains an uncut control from a reaction run with $CaCl_2$ (blue Ca^{2+} label), and the location of cut and uncut products are marked with red arrows. The full 22bp DNA sequences of the seven alternative target sites are listed to the right of the gel, with each single basepair change away from the original 17xSeq sequence highlighted in red. A magenta asterisk designates the target cleaved best by the aHE15 enzyme. **(B)** Acrylamide gel from a repeat of the experiment comparing the cleavage of all four single off-targets side-by-side with the original 17xSeqtarget and the seven combinatorial targets. A Ca^{2+} lane serves as an uncut control, and the location of cut and uncut products are marked with red arrows. **(C)** Quantification of the cleavage products from the gel shown in **Supplementary Figure S13b** with bars representing relative cleavage activity compared to the cleavage of the original target sequence (17xSeq). A dotted line designates the level of cleavage of the original 17xSeq target (a relative cleavage activity of 1.0.)
